# Population Pharmacokinetics and Pharmacokinetic-Pharmacodynamic Relationships of Methadone in a Sample of Iranian (Mazandarani) Opiate Users Undergoing Methadone Maintenance Treatment

**Published:** 2011

**Authors:** Mohammad-Reza Shiran, Rasa Hosseinzadeh, Abolhassan Hamidikenari, Mehran Zarghami, Nargess Lamsehchi, Mohammad-Reza Rafati

**Affiliations:** 1 Psychiatry and Behavioral Sciences Research Center, Faculty of Medicine, Mazandaran University of Medical Sciences, Sari, Iran; 2 Department of Pharmacotherapy, Faculty of Pharmacy, Mazandaran University of Medical Sciences, Sari, Iran

**Keywords:** Iranian, Methadone, Opiate Users, Pharmacodynamics, Pharmacokinetics

## Abstract

**Objective:** To investigate the pharmacokinetics (PK) and PK- pharmacodynamic (PD) relationship of methadone in a cohort of outpatients undergoing methadone maintenance treatment (MMT).

**Methods:** Sixty male patients undergoing MMT with a mean ±SD methadone daily dosage of 58 ± 34 mg were enrolled in this study. A 5-ml blood sample was collected before the daily intake of methadone. As a PD measure, the Subjective Opioid Withdrawal Scale (SOWS) form was completed immediately after obtaining the blood sample. Blood samples were taken and the forms were completed 4-5 times more (up to 24 hr) after the daily intake of methadone. Plasma methadone was analyzed using HPLC. Population PK/PD analysis was performed using population pharmacokinetics modeling software P-Pharm.

**Results:** Significant decreases (p< 0.05) were observed in the SOWS scores during 10 hours after methadone intake. The SOWS had returned to baseline by 24 hr after using methaodone (p= 0.98). A considerable interindividual variability in the CL/F (16 fold), EC_50 _(3 fold) and E_max _(6 fold) for methadone was observed.

**Conclusion:** Withdrawal symptoms were significantly improved in MMT patients after taking methadone and the PD measure was substantially affected by fluctuations in plasma methadone concentration. However, The SOWS had returned to baseline by 24 hr after using mathadone. Thus, a once daily dosing of methadone may not be suitable for those MMT patients who experience a significant withdrawal disturbance in the latter part of the interdose interval. This may increase the perceived severity of withdrawal and induce a craving for additional opioids.

## Introduction

Methadone is currently the preferred drug of choice for the treatment of opioid dependence and dominates the substitute opioid-prescribing market in Iran, accounting for perhaps 90% of it (1). A successful treatment leads to rehabilitation and socialisation of the dependent individual, reduction or elimination of taking illicit drug and its harmful consequences. The effectiveness of treatment is governed by the degree to which methadone prevents opioid withdrawal symptoms. Methadone doses have usually been adjusted on the conventional clinical parameters in methadone clinics in Iran. Even in MMT programmes associated with relatively high doses, some patients experience withdrawal symptoms for parts of the time between doses. There may be a pharmacokinetics and/or pharmacodynamics basis for these observations. Genetic polymorphisms in genes coding methadone-metabolising enzymes, transporter proteins, and µ-opioid receptors may explain part of the observed interindividual variation in the pharmacokinetics and pharmacodynamics of methadone (2).

The majority of information on the pharmacokinetic- pharmacodynamic relationships (PK/PD) of methadone comes from studies of patients with chronic pain (3,4), healthy subjects given a single oral dose of methadone (5), or from animal studies (6,7). However, there are limited studies of this type in MMT patients (8-11). It has been shown that PK/PD studies of opioid drugs can be hampered by factors such as the development of tolerance (12,13), and cross-tolerance phenomena (14). Therefore, the PK/PD relationships found after a single dose will not adequately predict those after a continuous exposure, and drug efficacy and/or drug potency are likely to be altered during the withdrawal because of a previous exposure to an opioid (14-16).

The aims of the present study were 1) to characterise the pharmacokinetics of methadone at steady-state, 2) to evaluate and compare the time profiles of withdrawal symptoms after methadone intake, and 3) to characterize the relationship between plasma methadone concentrations and its PD effect in a large cohort of Mazandarani ethnic group of Iranian Opiate users undergoing MMT.

## Materials and Methods

*Subjects*

Sixty male opioid dependent volunteers undergoing MMT for 9 ± 3 months with a methadone daily dosage of 58 ± 34 mg and age of 38 ± 8 years were recruited from Substance Misuse Clinic in Razi Hospital (Ghaemshar, Mazandaran, Iran) over a period of 6 months, each during a routine visit to the MMT clinic by the physicians involved in the study (convenience sampling). All participants were of Mazandarani origin residing in Mazandaran, a northern province in Iran. They were excluded from the study if they had known HIV, HBV, and HCV positive serology. All subjects gave written informed consent before recruitment and the research protocol was approved by the Research Ethics Committee of Mazandaran University of Medical Sciences, Sari, Iran.

*Sample collection*

A 5-ml blood sample was collected in sterile heparinized tubes before the daily intake of methadone. Blood samples were taken and the scales were completed 4-5 times more (up to 24 hr) after the daily intake of methadone. After centrifugation of blood samples for 5 min (1000 g), the plasma samples were transferred to separate sterile propylene tubes. The plasma samples were stored at-25°C pending assay.

*Measurement of the withdrawal symptoms*

As it has been demonstrated that addicts may still experience subjective symptoms in the absence of any observable signs of withdrawal (17), the Subjective Opioid Withdrawal Scale (SOWS) was completed immediately after taking the blood sample to assess opium withdrawal symptoms. This self-administered scale provides the patients with an opportunity to be involved in their care, and in assessing the severity of their withdrawal symptoms. SOWS contains 16 symptoms, intensity of which is being rated on a scale of 0 (not at all) to 4 (extremely) by the patients, and is demonstrated to be a valid and reliable indicator of the severity of the opiate withdrawal syndrome over a wide range of common signs and symptoms (17). This measure has been demonstrated to be related to plasma methadone concentration in methadone patients (18).

*Study medication and chemicals*

Methadone tablets were supplied by Tolid-Daro Company (Tehran, Iran). Methadone as the hydrochloride salt was a gift from the department of Drug and Food of Mazandaran University of Medical Sciences. All other chemicals and reagents were of *high-performance liquid chromatography* (HPLC) or analytical grade and were purchased by commercial suppliers.

*Chromatography*

Methadone was assayed by the HPLC method of Pham-Huy *et al* (19). In brief, the equipment comprised a model Kanaver HPLC pump (K-1001), a Kanaver UV detector (K-2600). The chromatographic system was interfaced with a PC using EZ-Chrom Elite software. The chromatographic separation of methadone was performed on a Luna C18 analytical column (3 µM particle size, 4.60 mm × 100 mm I.D.) (Phenomenex, Cheshire, UK) coupled to a security guard C18 pre-column (Phenomenex), using a mobile phase of acetonitrile: water (30:70 v/v, pH adjusted to 3.5 by ortophosphoric acid). The mobile phase was delivered at a flow-rate of 0.5 ml/min for 1-8 minutes and then was increased to 1 ml/min by the end of run (18 min). The limit of quantiﬁcation was 5 ng/ml, and the intra- and interday coefﬁcients of variation of the assays were <15%.

Plasma sample was treated according to the method of Pham-Huy *et al* (19) with some modifications. In brief, 1 ml of thawed sample was mixed with midazolam (50 µl of 10 µg/ml as internal standard), 200 µl of 1 M NaOH, 6 ml of *n*-hexane, and 200 µl of isoamil alcohol. The mixture was mixed for 1 min using a mixer and then, it was centrifuged at 1500g for 10 min. The organic phase was transferred to another 5 ml polypropylene tube and was back-extracted in 200 µl of 0.05M HCL by mixing vigorously for 1 min. After further centrifugation at 1500g for 10 min, the organic phase was aspirated and discharged and 80 µl aliquot was injected into the HPLC.

*Data analysis*

*Pharmacokinetic and* *pharmacodynamic* *analysis*

PK and PD analysis was carried out using population PK modeling software P-Pharm (P-Pharm., version 1.5. InnaPhase, Ceretil, France). Selection of the best model was based on the lowest value of the Akaike Information Criteria (AIC), visual inspection of residuals for systematic error and the predicted versus actual concentration plots. Initial estimates of the PK parameters were derived from values reported in the literature. Both one and two compartment classical linear models at steady- state with first-order input were investigated for PK analysis.

Population PK parameter values from the best model obtained during the PK analysis served as covariates for the PK/PD analysis (a two-stage PK-PD link model).

E=E0×1-E max×Cencen+EC50n

 Where C_e_ is the expected drug concentration in the effect compartment; E is the effect; E_max_ is the maximum effect; EC_50_ is the concentration giving 50% of the E_max_; and n is the sigmoidicity factor.

*Statistical analysis*

Statistical analysis was performed using SPSS for Windows (ver.10, SPSS Inc., Chicago, USA). Multiple linear regression analysis was used to reveal trends in the population data. For comparing more than two means, analysis of variance (ANOVA) was used. For comparing paired clinical data, a paired sample *t*-test or 2-tailed Wilcoxon matched pairs signed-rank test was used. For comparing non-paired clinical data, either an independent samples *t*-test or a Mann-Whitney U test was used. In all cases P< 0.05 was considered to be statistically significant.

## Results

All sixty subjects completed the study. In addition to methadone, three patients were taking other medications (clonazepam, lorazepam, and clonidine). The descriptive statistics for the dose and trough plasma concentrations (pre-dose) of methadone are shown in table 1. There was a statistically significant correlation between trough concentrations of methadone and methadone doses (r = 0.61, P< 0.001).

**Table 1:** Descriptive statistics for the dose and trough plasma concentration (pre-dose) of methadone in MMT patients studied

Parameters	Mean	SD	95% CI On the Mean		Min	Max	Fold change
			Lower Bound	Upper Bound			
dose (mg)	58	34	47	69	5	170	34
trough plasma concentration (pre-dose)	260.24	228.38	176.47	344.01	57.84	1212.33	20.96

*Pharmacokinetic analysis*

A two-compartment steady state PK model with first-order input, first-order distributional rate constants and first-order elimination provided a significantly better fit to the concentration-time profiles compared with other models. A heteroscedastic error model (1/y^2) was more appropriate for all analyses. A log-normal distribution best described the inter-subject variability in all population PK parameters.

**Figure 1:**
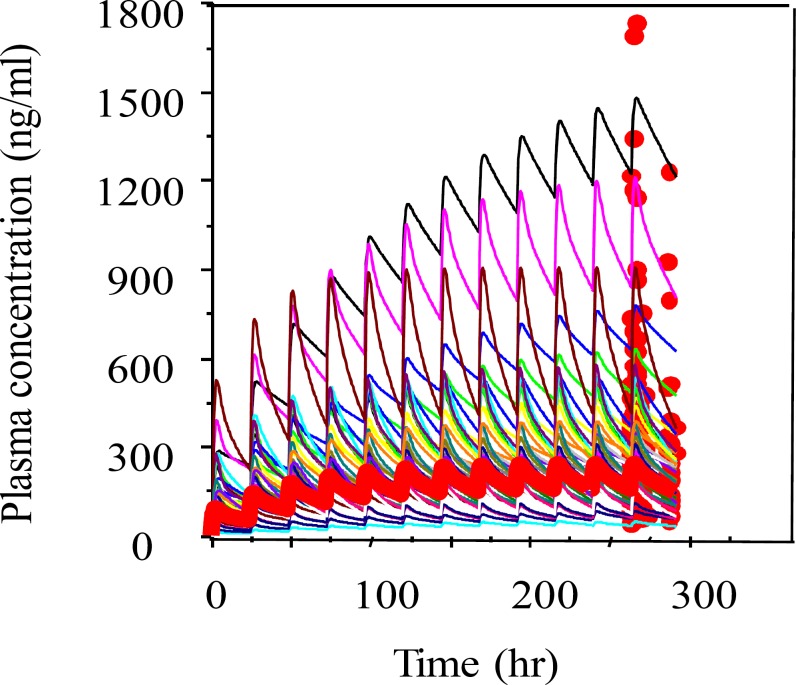
Population (bold line) and individual bayesian model fit to plasma concentrations of methadone at steady state.

**Figure2:**
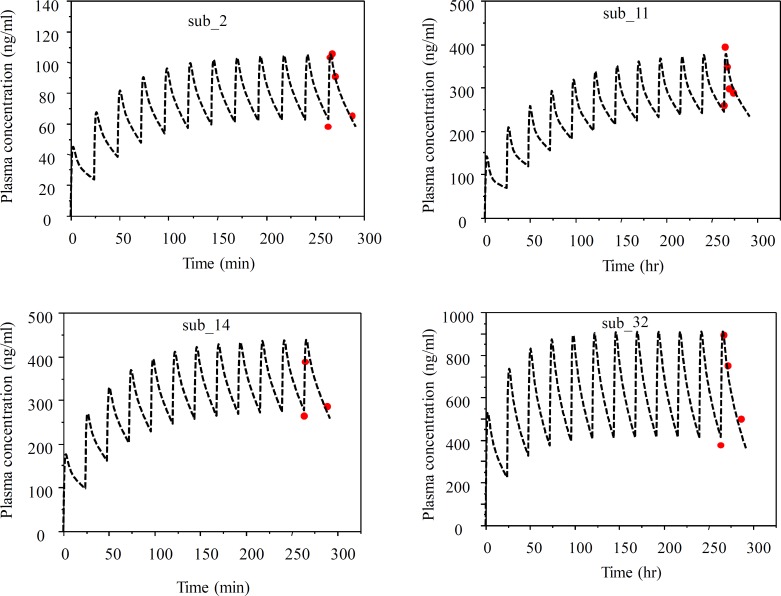
Representative individual Bayesian model fit to steady state methadone concentrations in patients with dosage from; 15 mg/day (C_ss_=80 ng/ml, sub-2), 60.mg/day (C_ss_=300 ng/ml, sub-11), 80.mg/day (C_ss_=360 ng/ml, sub-14), and 110 mg/day (C_ss_=650 ng/ml, sub-32).

Population mean and individual Bayesian model fit to methadone concentrations are shown in figure 1. Representative individual Bayesian model fit to steady state methadone concentrations at three different dosages are shown in figure 2.

Parameter values for methadone obtained from the best PK model are listed in table 2.

**Table 2:** Mean pharmacokinetic parameter values for methadone in MMT patients estimated from the best model.

PK parameters	Mean	SD	95% CI On the Mean		Min	Max	Fold changed
			Lower Bound	Upper Bound			
CL/F (L.h^-1^)	8.39	3.62	7.20	9.58	1.09	17.58	16.1
V/F (L)	131.10	28.41	121.76	140.44	64.04	201.00	3.1
t_1/2 _beta (h)	45.87	20.36	39.18	52.56	18.16	87.61	4.8
K12 (h^-1^)	0.57	0.04	0.55	0.58	0.46	0.69	1.5
K21(h^-1^)	0.25	0.05	0.23	0.26	0.15	0.41	2.7
Ka (h^-1^)	0.43	0.08	0.40	0.45	0.30	0.72	2.4

*Pharmacodynamic analysis*

*Model independent analysis*

**Figure 3:**
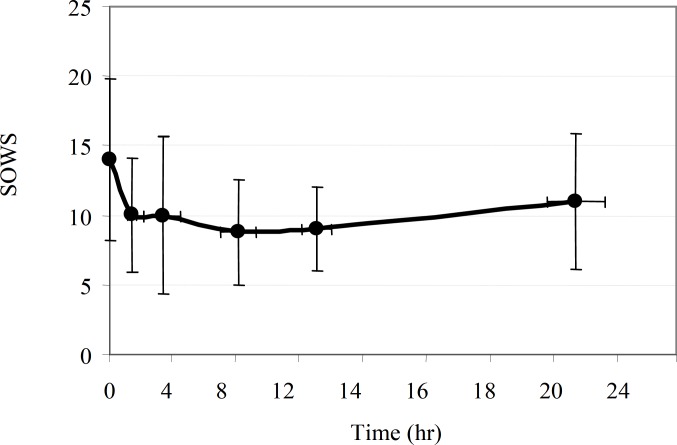
Comparisons of the time profiles of SOWS in MMT patients before (time = 0) and following methadone intake. The circles represent the mean values and the bar lines indicate 95% CI on the mean. A significant decrease (p ≤ 0.05) was observed in SOWS for 10 hr after methadone intake. The SOWS had returned to baseline by 24 hr after dose (p = 0.34).

The mean of SOWS comparisons of their time profiles in MMT patients before and following daily methadone intake are shown in figure 3. A significant decrease (p≤0.05) was observed in SOWS during 10.hr after using methadone in MMT patients, whereas the SOWS had returned to baseline by 24 hr after it (p = 0.34). 

*PK/PD modeling analysis*

**Figure 4:**
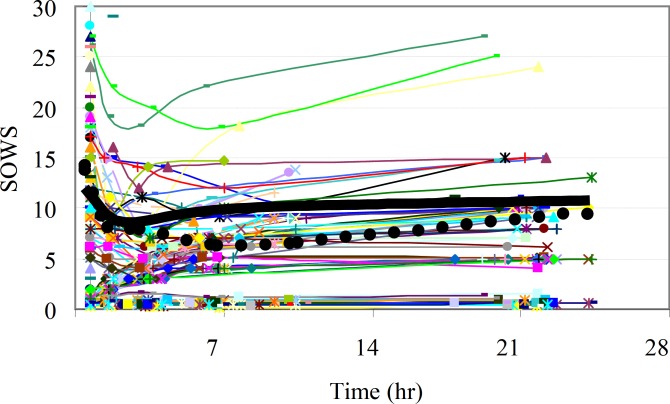
Observed (symbols) individual score with its mean values (dashed line) and the Population predicted score (black solid line) with respect to SOWS time profile by the best model fit for methadone.

Plasma methadone concentration-effect relationships were determined for the SOWS. The relationship between the changes in observed PD effect data SOWS and plasma concentration of methadone showed a time delay resulting in a counter-clockwise hysteresis loop. Thus, an effect compartment was assumed in all PK-PD models. Sigmoid E_max _model with effect compartment provided a better fit to the SOWS-time profiles for most analysts as determined by criteria discussed before. The individual SOWS with population mean values and population predicted values according to best model fit for methadone are presented in figure 4.

The EC_50_ and Emax of methadone with respect to SOWS showed considerable interpatient variability (Table 3).

**Table 3:** Population pharmacodynamic parameters for methadone with respect to SOWS estimated from the best model.

PD parameters	Mean	SD	95% CI On the Mean		Min	Max	Fold change
			Lower Bound	Upper Bound			
EC50(ng/ml)	413.47	108.97	377.65	449.29	218.18	632.12	2.90
Emax (%)	31.98	12.37	27.92	36.05	11.85	78.47	6.62

## Discussion

This is, to our knowledge, the first study characterizing the population pharmacokinetics of methadone in a sample of opiate users in Iran.

*Pharmacokinetics*

Methadone displays a wide dose–plasma concentration relationship and the administered dosage is an important determinant of methadone blood concentration. In order to obtain methadone plasma concentrations of 250 ng/ml in a 70-kg patient without any co-medication, doses of methadone as low as 55 mg/day or as high as 921 mg/day can be required (2). The results of the present study show that the correlation between dose and trough concentrations of methadone is statistically significant. These findings are consistent with a previous report (20). Several studies have aimed to find the optimum trough plasma methadone concentration for effective maintenance therapy (9,21-23). In some studies values ranging from 50 to 600 ng/ml of methadone have been proposed (21, 23-26). However, a concentration of at least 400 ng/ml of trough plasma methadone is now often considered necessary to provide stabilised maintenance, and is used as the reference concentration for therapeutic drug monitoring. The mean of trough plasma concentration of methadone in the present study (260.24 ± 228.38) was significantly (p<0.001) less than the proposed reference concentration.

The rate of oral clearance of methadone has been reported to be relatively variable with values ranging from 2 to 20 (L.h^-1^). Steady-state oral clearance of methadone in present study was in the range of values reported in previous studies (27). A considerable interindividual variability (16 fold) in the CL/F for methadone observed in the present study may be due to interindividual variability in the activity of the CYP enzymes and transporter proteins involved in the first pass metabolism and systemic clearance of methadone (2, 28, 29). The apparent volume of distribution and elimination half-life of methadone in present study are comparable with the findings from previous reports (27).

*Pharmacodynamic* *s*

In agreement with previous reports (8,18,30), this study has confirmed that withdrawal is significantly changed after methadone intake and strongly reflects fluctuation in plasma methadone concentration. A significant decrease was observed in SOWS during 10 hr after methadone intake in our MMT patients. However, the SOWS had returned to the baseline by 24 hr after methadone intake (p = 0.34). Although the confidence limit for SOWS are too wide to draw definitive conclusions, it is likely that a once daily dose of methadone may not be adequate to improve SOWS disturbances in some patients in present study and this may increase the perceived severity of withdrawal and induce a craving for additional opioids. These findings may have several important clinical implications. Once-daily dosing of methadone among the study participants (58 ± 34 mg/day) may not be suitable for those MMT patients who experience a significant SOWS disturbance in the latter part of the inter-dosing interval. An increase in the dose to 60-100 mg/day (31) and dividing the methadone dose into twice-daily administration may be a possible strategy.

Majority of information on PK/PD relationships for methadone come from studies of patients with chronic pain (3, 4) and in healthy subjects given a single oral dose of methadone (5). However, there are limited studies of this type in MMT patients (9, 11, 32). The calculated EC50 of methadone for SOWS in the present study was in the range of previously reported for pain relief (3, 4). However, the differences between the two populations and the study designs make comparison difficult. Inturrisi *et al* studied the PK/PD relationship for analgesia response in a small group of patients who had been receiving opioids other than methadone for chronic pain both after a single i.v bolus (3) and infusion (4) of methadone. The mean estimated values of EC50 were 290 ng/ml and 360 ng/ml respectively. It was shown that the pharmacodynamic relationship found after a single drug administration is not adequate to describe the pharmacodynamics of methadone after continuous exposure. This is possibly due to acquired tolerance.

The estimated EC50 for methadone with respect to the SOWS in the present study (414 ng/ml) was also in the range of previously reported values for withdrawal scores and pupil size in MMT patients (9, 32). In the Dyer *et al* (8, 9) studies, the estimated EC50 with respect to withdrawal scores and pupil size were 300 and 280 ng/ml respectively. The mean of the estimated EC50 with respect to withdrawal scores in the present study was significantly (p<0.001) less than those reported in Dyer studies (8, 9).

The high interindividual variability (6 fold) in the Emax of methadone observed in the present study might be explained by genetic polymorphisms in genes coding µ-opioid receptors or changes at the µ-opioid receptors. Diaz et al (33) have reported down-regulation of the µ-opioid receptor after a continuous exposure to an opioid. The decreased intrinsic activity of methadone after a chronic treatment with morphine is consistent with down-regulation when the receptor reserve is limited (34). Genetic polymorphisms have been characterized for genes encoding μ-opioid receptors (2, 35) and G proteins (36), all of which modulate response to methadone therapy independent of dose and exposure levels. These may also explain part of this variability. The clinician must be aware of the PK and PD properties of methadone in order to personalize methadone administration.

## Authors' Contributions

MR Sh conceived and designed the evaluation, performed the PK, PD, and statistical analysis, interpreted the clinical data and drafted the manuscript. RH participated in designing the evaluation and performed parts of the clinical data collection, PK, PD, and statistical analysis. AH re-evaluated the clinical data, and PK, PD, and statistical analysis. MZ participated in designing the evaluation and revised the manuscript. NL and MR R participated in clinical data collection, PK, PD, and statistical analysis. All authors read and approved the final manuscript.

## Notes

**Declaration of Interest:** None.
